# Case series of the first five human infections with monkeypox virus clade Ib and report on the public health response, United Kingdom, October to November 2024

**DOI:** 10.2807/1560-7917.ES.2025.30.10.2500131

**Published:** 2025-03-13

**Authors:** Muhammad Ibaad Alvi, Merav Kliner, William Welfare, N Claire Gordon, Sherine Thomas, Simon Padfield, Hannah E Emmett, Ellen Heinsbroek, Gareth J Hughes, Natalie Groves, Eileen Gallagher, Steven Pullan, Amy Belfield, Catherine F Houlihan, Tommy Rampling, Geraldine O’Hara, Anne Tunbridge, Jake Dunning, Elizabeth Whittaker, Alejandra Alonso, Mike Beadsworth, Brendan AI Payne, Meera Chand, Susan Hopkins, Gillian Armstrong

**Affiliations:** 1 United Kingdom Health Security Agency on behalf of the UKHSA IMT, London, United Kingdom; 2 Sheffield Teaching Hospitals NHS Foundation Trust, Sheffield, United Kingdom; 3 Royal Free London NHS Foundation Trust, London, United Kingdom; 4 Guy’s and St Thomas’s NHS Foundation Trust, London, United Kingdom; 5 Imperial College Healthcare NHS Trust, London, United Kingdom; 6 Evelina London Children’s Hospital, London, United Kingdom; 7 Liverpool Foundation Trust, on behalf of the NHS Airborne HCID Network, Liverpool, United Kingdom; 8 Newcastle upon Tyne Hospitals NHS Foundation Trust, Newcastle, United Kingdom; 9 University College London, London, United Kingdom; 10 University College London Hospital NHS Foundation Trust, London, United Kingdom; 11 National Institute for Health and Care Research (NIHR) University College London Hospitals Biomedical Research Centre (BRC), London, United Kingdom

**Keywords:** mpox, contact tracing, clade Ib, orthopox, monkeypox virus

## Abstract

We report two importations of monkeypox virus clade Ib infection to the United Kingdom in 2024. The first was a traveller returning from Tanzania, Rwanda and Uganda, the second from Uganda. Both presented with fever and typical skin lesions; 147 contacts were followed up, 19 vaccinated with MVA-BN. Three household contacts of the first individual, including two children, became infected. These are the first reported autochthonous transmissions of clade Ib in Europe, and first paediatric cases outside the African continent.

On 14 August 2024, the World Health Organization (WHO) declared a public health emergency of international concern (PHEIC) in response to the upsurge of cases caused by monkeypox virus (MPXV) clade Ib in a number of countries in Africa [1]. Since then, exportation of cases has been reported outside of the affected areas to other continents [2-7]. Here, we report on the clinical features and epidemiological characteristics of the first five mpox cases infected with clade Ib virus in the United Kingdom (UK) and findings of public health relevance from the management of these cases.

## Mpox case detection

In the UK, mpox caused by MPXV clade I (Ia and Ib) is considered a high consequence infectious disease (HCID) [8,9]. All confirmed mpox cases are considered for admission to specialist national health service (NHS) centres for observation and isolation. Case 1 presented at the end of October 2024 with three household contacts (Cases 2, 3 and 4) who tested positive within 7 days. Case 5 presented at the end of November 2024.

### Case 1

**Figure 1:**
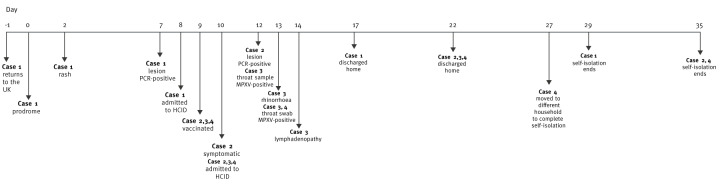
Timeline of symptoms and management the first four mpox cases with clade 1b infection, United Kingdom, October–November 2024

HCID: high consequence infectious disease; MPXV: monkeypox virus; UK: United Kingdom.

A male in his 40s returned to the UK following travel to Tanzania, Rwanda and Uganda in October 2024. During the trip, he reported having a massage and social contacts, but no other defined close contact exposures. On arrival to the UK, he was asymptomatic, but the next day developed a prodrome, and 2 days later developed skin lesions on the arms, face and chest (see the timeline in Figure 1). Seven days after symptom onset, he sought medical attention, and mpox testing was undertaken. A lesion swab was PCR-positive for MPXV [10] (quantification cycle (Cq) 20.4) and rapid clade determination was made, based on the recently published Ib-specific assay (dD14–16) [11] and the absence of the C3L target. This led to a 9-day stay in an airborne HCID treatment centre for isolation and clinical observation. He then continued to self-isolate at home until deemed non-infectious by clinical criteria. Whole genome sequencing confirmed clade Ib and relatedness to the East African outbreak (GenBank accession PQ628240.1).

Contact tracing identified three close household contacts (high-risk contacts), 13 community contacts from an outdoor seated event and nine healthcare contacts. High-risk contacts (see definition below) were quarantined for 21 days from last exposure to the case. Post-exposure vaccination with the modified vaccinia Ankara (MVA-BN; Bavarian Nordic) was accepted by seven of eight contacts offered (three high-risk, five medium-risk). Ring vaccination was offered to three household contacts of a high-risk contact, but all declined. Several co-travellers were identified; they did not meet the case definition for contacts and reported no symptoms.

All three close household contacts became infected (Cases 2, 3, 4). No contacts outside the household setting became infected.

### Case 2

Case 2 was the partner of Case 1, who quarantined when identified as a high-risk contact and received MVA-BN vaccine 10 days after first exposure. She developed a sore throat and lesions on the face and torso the day following vaccination. She was admitted to an airborne HCID treatment centre where a skin swab was PCR-positive for MPXV clade Ib (Cq 23). A disseminated rash developed. She remained in the hospital for 12 days, and completed isolation until lesions resolved at home. No additional contacts were identified during the follow-up period, other than the additional cases (3 and 4) already identified as contacts of Case 1. A sequence was not obtained for Case 2.

### Cases 3 and 4

Cases 3 and 4 were children of Cases 1 and 2, aged < 5 and 5–15 years, respectively. They were quarantined when identified as high-risk contacts and received MVA-BN 10 days after first exposure. The children were admitted to an airborne HCID treatment centre with Case 2. Case 3 developed rhinorrhoea and posterior cervical lymphadenopathy on day 4 and 5 post-vaccination, respectively. Both symptoms resolved within 48 h. A throat swab and urine sample tested positive for MPVX clade Ib on two occasions (Cq 36.4 and Cq 29.5, respectively) within the context of repeated testing. Case 4 remained clinically well throughout hospital stay with a small papule noted on the chin that was present for 24 h on day 7 post-vaccination. Case 4 had positive throat and skin swabs for MPXV clade Ib (Cq 36.2 and Cq 34.1, respectively). Cases 3 and 4 were discharged after 12 days following clinical resolution in Case 3 and negative swabs. No additional contacts were identified from Cases 3 or 4 during the follow-up period. Case 4 moved to a different household to complete self-isolation 15 days after hospital discharge.

### Case 5

**Figure 2:**
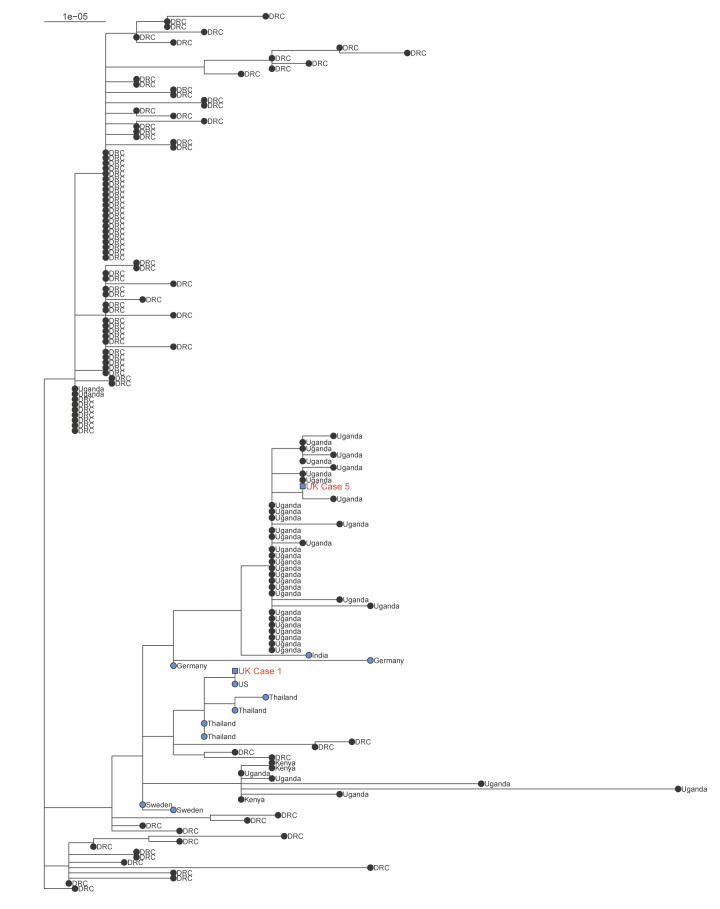
Maximum likelihood phylogenetic tree of sequences from mpox Cases 1 and 5, United Kingdom, and publicly available clade Ib sequences available up to 5 December 2024 (n = 150)

DRC: Democratic Republic of the Congo; UK: United Kingdom; US: United States.

Case 5 was a male in his 20s with recent travel to Uganda. During travel, he had social contact with friends and family, and heterosexual contact. During his trip, he developed a genital rash. Ten days later, he developed fever and received empirical treatment for malaria while still in Uganda. He returned to the UK while symptomatic. Six days following onset of fever, widespread lesions appeared. A lesion swab was PCR-positive for MPXV clade Ib (Cq 19.5) leading to admission to an airborne HCID unit. MPXV clade Ib was confirmed through whole genome sequencing, with the viral genome clustering phylogenetically with other genomes from Uganda (GenBank accession PQ 62586; Figure 2).

Sequences were obtained from GISAID [16] (n = 68) and GenBank (n = 82) available up to 5 December 2024. Sequences are labelled by country of origin. Cases outside of the African continent are indicated by blue tips. UK Case 1 and Case 5 are labelled in red. The tree was generated using Squirrel version 1.0.12, including masking of poor-quality positions recommended by the software, and visualised using ggtree version 3.6.2.

Contact tracing identified four household (high-risk) contacts who shared domestic spaces but had minimal direct interaction, 14 healthcare contacts, 59 domestic travel contacts, 31 flight contacts, and 14 community contacts. All four household contacts and five of 14 other higher risk contacts accepted post-exposure vaccination. No secondary cases were identified. 

## Management of contacts

The UK Health Security Agency (UKHSA) approach to contact classification and management, and numbers of contacts associated with all cases, are summarised in the Table and in line with published guidance [12]. In total, 147 contacts were followed up. Three high-risk contacts became cases (Cases 2, 3 and 4), and no medium- or low-risk contacts became cases. Sixteen of 29 contacts accepted the offer of post-exposure vaccination.

**Table:** Monkeypox virus clade Ib contact classification and management by the UK Health Security Agency, United Kingdom, October–December 2024

Exposure risk	Description	Contact management	Number of contacts (number vaccinated)	Number of cases
High (category 3)	Unprotected direct contact or high-risk environmental contact.	Active monitoring, self-isolation, avoid travel for 21 days. Offer MVA-BN vaccine within 4 days^a^.	7 (6)	3
Medium (category 2)	Unprotected exposure to infectious materials, droplet, or airborne potential.	Active monitoring for 21 days, risk assessment for work and/or education, avoid international travel. Offer MVA-BN within 4 days^a^.	24 (10)	0
Low (category 1)	Protected physical or droplet exposure, or no physical contact with unlikely droplet exposure.	Passive monitoring. Continue routine activities and travel if asymptomatic.	116 (0)	0
Total			147 (16)	3

MVA-BN: modified vaccinia Ankara.

^a^ Up to 14 days for high-risk groups like immunosuppressed individuals, pregnant women, or children aged under 5 years.

## Discussion

A highly precautionary approach was taken to the management of the first five individuals with mpox in the UK infected with MPXV clade Ib. Clade I (Ia and Ib) infections are considered a HCID in the UK, and managed with HCID protocols in healthcare settings. This includes stringent infection prevention and control protocols, and consideration of admission of all confirmed cases to specialist NHS centres for observation and isolation. Nearly 150 contacts were followed up, with transmission being detected only within a familial (close contact) household setting, and no transmission identified in healthcare or community contacts. This is consistent with a notable amount of close contact being required for transmission [13].

The two paediatric cases were pauci-symptomatic and only detected on repeated testing. It is therefore likely that in other settings such cases could remain unrecognised. The minimal symptomatology may be due to a naturally mild course of infection, transient carriage, or to vaccine-attenuated illness, noting that it would be early for a vaccine-mediated effect.

Just over half (16/29) of high- and medium-risk contacts who were offered post-exposure or ring vaccination accepted. Reasons given for refusal were low perception of risk, logistical barriers to accessing vaccine, needle phobia and anti-vaccination views. For a successful ring vaccination programme, greater uptake of vaccine would be required [14]. Action has been taken to streamline clinical pathways for access to timely post-exposure MVA-BN vaccination in England.

A number of healthcare contacts were identified, with greatest exposure (category 3) in healthcare assistants and cleaners [15]. This highlights the importance of education and training to support safe working practices in the wider care team, especially in clinical settings who see undifferentiated rash illness.

A coordinated multi-agency response involving UKHSA, NHS England and regional health protection teams was required to manage these cases and follow up contacts for 21 days, highlighting the resources required to manage such cases within public health and healthcare.

## Conclusion

This report describes the management of the first reported cases of mpox with clade Ib in the UK, including an autochthonous transmission event and the first paediatric cases reported outside of the African continent. The level of contact required for transmission of clade Ib MPXV in this small case series supports existing literature suggesting close contact is required for human-to-human transmission. As two of the cases, both children, had minimal symptoms and were only detected by repeated pauci-symptomatic testing, further research is needed to understand the role of asymptomatic and pauci-symptomatic cases in transmission of clade Ib.

## Supplementary Data

20000 Supplement
