# Supplementary material for: Case series of the first five human infections with monkeypox virus clade Ib and report on the public health response, United Kingdom, October to November 2024
Source: Euro Surveill. 2025 Mar 13;30(10):2500131. doi: 10.2807/1560-7917.ES.2025.30.10.2500131 (PMC11912141; doi:10.2807/1560-7917.ES.2025.30.10.2500131)
Supplement: Supplement [file 25-00131_ALVI_SUPPLEMENTAL_TABLE.pdf]

SUPPLEMENTAL TABLE

Data Availability

GISAID Identifier:	EPI_SET_250311wc
DOI:	https://doi.org/10.55876/gis8.250311wc

All genome sequences and associated metadata in this dataset are published in GISAID's EpiCoV database. To view the contributors of each individual sequence with details such as accession number, Virus name, Collection date, Originating Lab and Submitting Lab and the list of Authors, visit 10.55876/gis8.250107tk

Data Snapshot

EPI_SET_250311wc is composed of 68 individual genome sequences.
The collection dates range from 2023-11-02 to 2024-10-27;
Data were collected in 7 countries and territories.
